# Supplementary material for: Content-rich biological network constructed by mining PubMed abstracts
Source: BMC Bioinformatics. 2004 Oct 8;5:147. doi: 10.1186/1471-2105-5-147 (PMC528731; doi:10.1186/1471-2105-5-147)
Supplement: Additional File 2 — The original results of the above study (non-essential files are deleted to keep the file size under the limit set by BMC bioinformatics). [file 1471-2105-5-147-S2.bz2 › chilibotAdditionalFile2/dip20/562ID11502794E10923/html/IGF2_IGF2R.html]

 


 **IGF2** and **IGF2R** 
  
Found 192 abstracts in PubMed, retrieved 20.  
 

 What does Google say? 
 PDF only 
| .edu only 

---

**Interactive relationship** (e.g. stimulation, inhibition, etc)

**Neutral relationship**- LNCaP cells expressing a mutant  **IGF2R**  incompetent to bind IGF II  [ **IGF2** ]  IGF2RMyc I T proliferated more rapidly than both vector transfected cells and cells expressing the IGF2RMyc.  Ref: 12586773 Endocrinology, 2003
- The IGF II  [ **IGF2** ]  mannose 6 phosphate Man 6 P receptor  **IGF2R**  binds IGF II  [ **IGF2** ]  and Man 6 P bearing ligands at distinct binding sites.  Ref: 12586773 Endocrinology, 2003
- The subcellular distribution and ability to internalize cell surface IGF II  [ **IGF2** ]  of IGF2RMyc were indistinguishable from endogenous  **IGF2R**  in PC 3 cells.  Ref: 12586773 Endocrinology, 2003

**Non-interactive relationship** (e.g. studied together, co-existance, homology, etc.)

- These data suggest that the IGF II  [ **IGF2** ]  and Man 6 P binding functions of the  **IGF2R**  have opposing activities, with respect to growth of prostate cancer cells.  Ref: 12586773 Endocrinology, 2003
- Familial aggregation of abnormal methylation of parental alleles at the  **IGF2**  H19 and  **IGF2R**  differentially methylated regions.  Ref: 12812984 Hum Mol Genet< MedlineTA>Hum Mol Genet, 2003
- BACKGROUND Loss or mutation of the mannose 6 phosphate insulin like growth factor II  [ **IGF2** ]  receptor M6P  **IGF2R**  has been found in breast cancer.  Ref: 12149131 BMC Cancer, 2002
- We examined allelic methylation ratios at DMRs within the  **IGF2**  H19 and  **IGF2R**  loci in a panel of 48 three generation families.  Ref: 12812984 Hum Mol Genet< MedlineTA>Hum Mol Genet, 2002
- The  **IGF2**  receptor  **IGF2R**  is a USF2 specific target in nontumorigenic mammary epithelial cells, but not in breast cancer cells.  Ref: 12857727 J Biol ChemJ Biol Chem, 2002
- Our purpose was to evaluate inherited short tandem repeat polymorphisms of the insulin like growth factor II  [ **IGF2** ]  receptor gene  **IGF2R**  in oral cancer risk.  Ref: 12543777 Cancer Res, 2003
- In addition, we have extended physical maps of sheep and goat as 11 genes HSD3B1, INHBA, CSN10,  **IGF2R** , PIGR, MAP1B, DSC1, ELN, TNFRSF6, CGN1,  **IGF2**  and 14 genes SOD1, HSD3B1, CSN10,  **IGF2R** , RB1, TG, PIGR, MAP1B, IGH@, LTF, DSC1, TNFRSF6, CGN1,  **IGF2**  were assigned for the first time to goat and sheep chromosomes, respectively.  Ref: 12873218 Anim Genet, 2003
- Since the mannose 6 phosphate insulin like growth factor II  [ **IGF2** ]  receptor M6P  **IGF2R**  plays a central role in the intracellular transport and endocytosis of M6P containing lysosomal enzymes, a deficiency in functional M6P  **IGF2R**  may underlie the hypersecretion of procathepsin D in invasive tumors.  Ref: 12165733 Med Sci Monit, 2002
- We showed reliable and reproducible results in distinguishing overall methylation profiles of DMRs regions of human SNRPN, H19, MEST PEG1, LIT1,  **IGF2** , TSSC5, WT1 antisense, and mouse H19, Mest Peg1,  **Igf2R**  imprinted genes.  Ref: 12613258 Biotechniques, 2003
- Moreover, analysis of imprinted gene expression in a methyltransferase knockout Dnmt1 mouse has shown that the imprint is lost in a number of cases, resulting in either two silent alleles  **Igf2** ,  **Igf2r**  and Kvlqt or two expressed alleles H19, p57kip2, Snrpn, and Xist 0 12.  Ref: 12843456 Methods Mol Biol, 2001
- At  **Igf2r**  encoding insulin like growth factor 2  [ **IGF2** ]  receptor, a sequence called region 2 is needed for methylation of the active maternal allele.  Ref: 12740578 Nat Genet< MedlineTA>Nat Genet, 2001
- To determine when in germ cell development this biallelic expression phenomenon commences, we isolated migrating PGCs by flow cytometry and determined the allele specific expression of four imprinted genes Snrpn,  **Igf2** , H19 and  **Igf2r** .  Ref: 12049782 Mech Dev, 2002
- Previous studies screening for quantitative trait loci QTLs that influence general intelligence in healthy individuals have identified four loci, two of which are located within the genes insulin like growth factor 2  [ **IGF2** ]  receptor  **IGF2R**  and the Msx1 homeobox.  Ref: 12556904 Mol Psychiatry, 2003
- Furthermore, decreased expression of M6P  **IGF2R**  enhanced IGF II  [ **IGF2** ]  induced proliferation and reduced cell susceptibility to TNF induced apoptosis.  Ref: 12149131 BMC Cancer, 2002
